# Supplementary material for: COVID-19 treatment combinations and associations with mortality in a large multi-site healthcare system
Source: PLoS One. 2021 Jun 11;16(6):e0252591. doi: 10.1371/journal.pone.0252591 (PMC8195431; doi:10.1371/journal.pone.0252591)
Supplement: Attachment — Submitted filename: Response to Reviewers.pdf [file pone.0252591.s001.pdf]

Jefferson Health

Department of Medicine
Division of Infectious Diseases

1015 Chestnut Street, Suite 1020
Philadelphia, PA 19107
T 215-955-7785
F 215-955-9362

Nana Aburjania, MD
Katherine A. Belden, MD
Caroline C. Brugger, CRNP
Rachel Carr, PA-C
Dagan Coppock, MD
Joseph A. DeSimone, MD
Phyllis Flomenberg, MD
Elizabeth Gancher, MD
Sarah E. Green, CRNP-BC
Molly Hancock, PA-C
Bryan Hess, MD
Carolyn Kramer, MD
Sean Moss, MD
Tricia Royer, DO
Lisa A. Spacek, MD, PhD
Kathleen Squires, MD
Gustavo Vasquez, MD
Devin Weber, MD
John Zurlo, MD

April 12, 2021

To the Editors of PLOS ONE:

We are resubmitting the manuscript "COVID-19 Treatment Combinations and Associations with Mortality in a Large Multi-Site Healthcare System" (Manuscript Number PONE-D-20-35523) for further review. Reviewer comments have been incorporated into the revised manuscript. We have itemized the comments and our responses to those comments below. Further, we have placed a de-identified dataset in the Harvard Dataverse repository at: https://doi.org/10.7910/DVN/DO66ZR.

Reviewer #1:

Comment: 1. One concern I have is whether assumptions of logistic regression were met. Please clarify how you validated the assumptions of logistic regression. It's important that the data used meet major assumptions of logistic regression for the analysis to be valid.

Response: Major assumptions of logistic regression were met, including the use of binary dependent variables, lack of multicollinearity (variance inflation factor below 2) and independence of errors (data came from different patients). This is now addressed in the Results section of the manuscript (see paragraph starting at line 170).

Comment: 2. In the abstract, the author says that 'multivariate logistic regression' was used, but 'multivariate' and 'multivariable' are two distinct approaches. In the results section the author says that "for all patients, four treatment combinations were associated with lower mortality: …, and anticoagulation, corticosteroids and remdesivir (OR 0.42, p <0.026)." I believe the author meant to say (OR 0.42, p=0.026).

Response: Thank you for this great point. Multivariable logistic regression is the correct terminology. We have revised the manuscript accordingly. Regarding the p-value, thank you for detecting our error. The results now read OR 0.42, p=0.026 instead of OR 0.42, p <0.026.

Comment: 3. In line 35, the authors report that 'In the United States, mortality related to COVID-19 has been on the decline. This may be due to a change in treatment strategies, a change in patient characteristics, or a combination of both." A reference/evidence to support this statement might be helpful. Does the data used for the study support shift in treatment patterns as well as shift in patient demographics?
Response: References are now provided to support the above statement. Furthermore, like other institutions, we have observed a shift in treatment patterns and patient demographics during the Covid-19 pandemic. A statement related to this is now included in the revised discussion section (see line 243).

Comment: 4. Table 1 reports the mean/percentage of patient demographics and clinical characteristics. Reporting a measure of variability (standard deviation or standard error of the mean) for inpatient length of stays (days) or showing a general distribution of the length of stay might be helpful. The author may also recode patient length of stay as a categorical variable.
Response: We have now provided the standard deviation for length of stay stratified by outcome in the revised Results section.

Comment: 5. Table 4 reports the differences in mortality between the two time periods. I thought a figure showing variability over time would serve this paper well, especially in showing outcome changes over time (showing confounding). Again, a measure of variability (confidence interval or standard deviation) might be helpful in understanding the results.
Response: Table 4 has been revised. The observed mortality rate for the two time periods is now included (i.e., the non-risk adjusted mortality is now included). When comparing two time periods (versus repeated measurements), confidence intervals are not provided unless bootstrapping is performed. Since our study was a retrospective empirical study, we did not feel bootstrapping was necessary.

Comment: 6. How was the model validated? Reporting a goodness of fit statistic (for example, the Hosmer Lemeshow goodness of fit test) might be helpful in evaluating how well the proposed model fits or predicts a set of observations.
Response: Because the purpose of our study was to identify correlated risk factors, not to predict mortality, we did not perform training and holdout validations. However, Hosmer and

Lemeshow tests did show a good fit to our model and a statement related to these results is now included in the revised manuscript (see line 174).

Reviewer #2:

Comment: (1) Was disease severity (mild, moderate, severe, critical) divided among the group? I assumed most hospitalized patients had the severe or critical disease. However, in-hospital transmission cases or patients with other comorbid conditions might have been admitted for the mild or moderate disease. One must be cautious looking into the mortality as critical COVID patients may receive certain drugs versus mild COVID patients may not receive other than a routine stand of care prophylactic anticoagulation but have better outcomes.

Response: This is an excellent point. To address this issue, we performed multivariable regression analyses on both ICU and non-ICU patient populations. We have edited our Methods section (see line 114) to clarify this approach.

Comment: (2) Over 90% received anticoagulation in COVID 19 patients. Were there specific criteria for anticoagulation use?

Response: The decision to use anticoagulation was a combination of institutional thromboprophylaxis guidelines and individual provider discretion. A statement related to this is now included in the Discussion section of the revised manuscript (see line 281).

Comment: (3) Regarding the cause of death in the ICU group without anticoagulation, were there higher thromboembolic events?

Response: This study did not explore this endpoint. However, other investigators at our institution are currently examining the association between anticoagulation and thromboembolic events at Jefferson Health.

Comment: (4) Consider including recent Multi-platform trial data regarding anticoagulation in the discussion section.https://www.nih.gov/news-events/news-releases/full-dose-blood-thinners-decreased-need-life-support-improved-outcome-hospitalized-covid-19-patients

Response: We have now included this in the revised discussion (see line 282).

General Editor (from initial submission):

Comment: 1. Please ensure that your manuscript meets PLOS ONE's style requirements, including those for file naming. The PLOS ONE style templates can be found at https://journals.plos.org/plosone/s/file?id=wjVg/PLOSOne_formatting_sample_main_body.pdf and https://journals.plos.org/plosone/s/file?id=ba62/PLOSOne_formatting_sample_title_authors_affiliations.pdf

Response: The manuscript has been formatted to meet PLOS ONE’s style requirements.

Comment: 2. In the ethics statement in the manuscript and in the online submission form, please provide additional information about the patient records used in your retrospective study. Specifically, please ensure that you have discussed whether all data were fully anonymized before you accessed them.

Response: The ethics statement has been clarified to reflect that this project was evaluated by the Jefferson University Institutional Review Board and received a waiver of authorization for use of protected health information (control number 20E.846).

Comment: 3. In statistical methods, please refer to any post-hoc corrections to correct for multiple comparisons during your statistical analyses. If these were not performed please justify the reasons. Please refer to our statistical reporting guidelines for assistance (https://journals.plos.org/plosone/s/submission-guidelines.#loc-statistical-reporting).

Response: To address false significances from multiple testing, we calculated adjusted p-values by using (1) the step-down Bonferroni method and (2) the linear step-up method of Benjamini and Hochberg that that controls the false discovery rates. This is now stated in our Methods section (see line 120).

Comment: 4. In your statistical analyses, please state whether you accounted for clustering by hospital. For example, did you consider using multilevel models?

Response: Per common practice, we pooled patients across multiple facilities and did not perform multilevel modeling or fixed-random effect model. Identifying facility cluster effects was not one of our initial study objectives. However, it would be interesting to explore the hospital effects and understand how patient and treatment risks can be different across facilities. We consider this a good further research topic.

Comment: 5. We note that you have indicated that data from this study are available upon request. PLOS only allows data to be available upon request if there are legal or ethical restrictions on sharing data publicly. For information on unacceptable data access restrictions, please see http://journals.plos.org/plosone/s/data-availability#loc-unacceptable-data-access-restrictions.
Response: A deidentified data set has been placed in the Harvard Dataverse repository. It may be reviewed at https://doi.org/10.7910/DVN/DO66ZR.

General Editor (from re-submission):
Comment: 1) Thank you for including your ethics statement on the online submission form: "This study was approved by the Jefferson University Institutional Review Board (protocol approval #20E.846). The study received a waiver for consent by the Jefferson University Institutional Review Board." To help ensure that the wording of your manuscript is suitable for publication, would you please also add this statement at the beginning of the Methods section of your manuscript file.
Response: We have moved the ethics statement to the beginning of the Methods section.

Comment: Thank you for your e-mail to PLOS ONE following our queries regarding data availability… In light of this we are unable to sign the DUA presented. Furthermore, we would be grateful if you could provide some clarification on the following:
2.1) Whether you are still able to make the data available in a public repository (preferred) or as part of the submitted article.
2.2) If there are restrictions to sharing the data as above, please indicate who is imposing these restrictions…
2.3) …and why such restrictions are imposed on the data.
Response: We have added the de-identified dataset to the Harvard Dataverse repository. There are no restrictions on sharing the de-identified data.

Thank you for your consideration of this revised manuscript. We hope these revisions address any concerns. Please contact me with any further questions.

Sincerely,

Dagan Coppock, MD

Dagan Coppock, MD
Thomas Jefferson University
Division of Infectious Diseases
(c) 203-915-7302
(o) 267-503-8845
(f) 215-503-3417
dagan.coppock@jefferson.edu
